# The influence of dose distribution on treatment outcome in the SCOPE 1 oesophageal cancer trial

**DOI:** 10.1186/s13014-016-0594-x

**Published:** 2016-02-06

**Authors:** Rhys Carrington, Emiliano Spezi, Sarah Gwynne, Peter Dutton, Chris Hurt, John Staffurth, Thomas Crosby

**Affiliations:** School of Medicine, Cardiff University, Cardiff, UK; School of Engineering, Cardiff University, Cardiff, UK; South West Wales Cancer Centre, Swansea, UK; Wales Cancer Trials Unit, Cardiff University, Cardiff, UK; Department of Clinical Oncology, Velindre Cancer Centre, Cardiff, UK

## Abstract

**Purpose:**

The first aim of this study was to assess plan quality using a conformity index (CI) and analyse its influence on patient outcome. The second aim was to identify whether clinical and technological factors including planning treatment volume (PTV) volume and treatment delivery method could be related to the CI value.

**Methods and materials:**

By extending the original concept of the mean distance to conformity (MDC) index, the OverMDC and UnderMDC of the 95 % isodose line (50Gy prescribed dose) to the PTV was calculated for 97 patients from the UK SCOPE 1 trial (ISCRT47718479). Data preparation was carried out in CERR, with Kaplan-Meier and multivariate analysis undertaken in EUCLID and further tests in Microsoft Excel and IBM’s SPSS.

**Results:**

A statistically significant breakpoint in the overall survival data, independent of cetuximab, was found with OverMDC (4.4 mm, *p* < 0.05). This was not the case with UnderMDC. There was a statistically significant difference in PTV volume either side of the OverMDC breakpoint (Mann Whitney *p* < 0.001) and in OverMDC value dependent on the treatment delivery method (mean IMRT = 2.1 mm, mean 3D-CRT = 4.1 mm Mann Whitney *p* < 0.001). Re-planning the worst performing patients according to OverMDC from 3D-CRT to VMAT resulted in a mean reduction in OverMDC of 2.8 mm (1.6–4.0 mm). OverMDC was not significant in multivariate analysis that included age, sex, staging, tumour type, and position.

**Conclusion:**

Although not significant when included in multivariate analysis, we have shown in univariate analysis that a patient’s OverMDC is correlated with overall survival. OverMDC is strongly related to IMRT and to a lesser extent with PTV volume. We recommend that VMAT planning should be used for oesophageal planning when available and that attention should be paid to the conformity of the 95 % to the PTV.

## Introduction

In the UK, oesophageal cancer is the sixth most common cause of cancer, accounting for around 5 % of all cancer deaths [1]. Long term survival for operable squamous cell carcinomas treated with definitive chemoradiotherapy (dCRT) is comparable to surgery alone [2], and is also more effective than either radiation therapy or chemotherapy alone [3, 4]. It is clear that radiotherapy (RT) now plays a key role in the treatment of these tumours, however the formulation and application of optimal RT protocols to these sites is not well defined [5]. There is a clear need however to improve the quality and outcome of therapy. It is known that dose distribution is an important factor when evaluating the quality of RT plans. A decisive parameter when considering the acceptability of a plan is whether 95 % of the prescribed (tumoricidal) dose is delivered to 100 % of the planning target volume (PTV) [6, 7]. However, although this requirement will be met in the majority of patients undergoing RT, the quality of the dose distribution may vary according to factors such as PTV volume [8] or by the delivery technique [9]. It has been shown that adherence to a site-specific RT protocol is effective in improving plan quality and patient outcomes [10], and the SCOPE 1 trial (a National Cancer Research Institute and Cancer Research UK funded Phase II/III two arm trial of dCRT with and without cetuximab in oesophageal cancer [11]) provided a detailed RT study protocol and quality assurance programme [12].

SCOPE 1 showed low rates of acute and late toxicity with 50 Gy in 25 fractions over 5 weeks concurrent with chemotherapy [13]. Two-year overall survival was 56 % in the control arm, higher than previously reported in published studies; However, no benfit was seen for the addition of Cetuximab in the experimental arm.

Despite a detailed radiotherapy protocol and planning guidance document, a rigorous Radiotherapy Treatment Trials Quality Assurance (RTTQA) programme [14, 15] demonstrated variation in RT planning practice such as planning technique across the 34 UK centres that participated in this study. These factors may have affected the quality of the dose distribution achieved for each patient, in addition to those mentioned previously. A recent study by Mutic et al. noted that many plans may still be classed as ‘low quality’ even when adhering to clinical trial protocol [16].

We hypothesised that plan quality can be objectively assessed by quantifying the relationship between the 95 % isodose and the PTV using a conformity index (CI) and explored the effect of variation in these measures on patient outcome (in this case the survival data from the SCOPE 1 trial). The use of conformity indices to analyse dose distribution of RT plans has been carried out previously [17, 18]. However, to the best of our knowledge, this study is the first to explore the relationship of CI and patient outcome.

The aim of this study was two fold; Firstly to assess plan quality using a CI and analyse its influence on patient outcome. Secondly, to identify whether clinical and technological factors including PTV volume and treatment delivery method could be related to the CI value.

## Materials and methods

### Study workflow

**Fig. 1:**
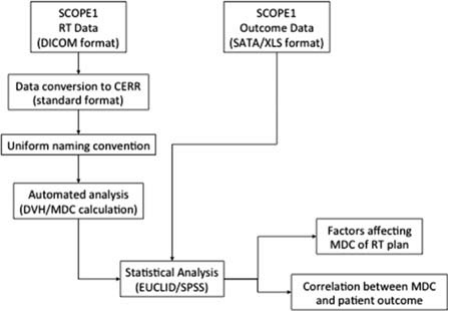
Workflow showing different stages of study

This study’s key stages are shown in Fig. 1. The following section gives an in depth account of how the data was gathered, a suitable database created and the analysis undertaken.

### Data preparation and analysis

SCOPE1 has been ethically approved by the Research Ethics Committee for Wales and has approval from the Medicines and Health Care Product Regulatory Agency to be conducted in the UK. The RTTQA programme for the SCOPE 1 trial required centres to submit 3D dose and structure data in DICOM RT format for of all patients treated within the trial to the Wales Cancer Trials Unit (WCTU). We imported and processed this data into the CERR software package (Computational Environment for Radiotherapy Research) [19]. Previous work from our institution found that the dose distribution within a RT plan is algorithm dependent [14] and that plan optimization carried out with algorithms that model lateral electron transport results in improved PTV coverage. These algorithms have been classified as “Type B”. Of the 206 patients in the SCOPE 1 trial, 97 were planned with Type B algorithms and form the dataset for this study.

We ensured the integrity of the database by checking that the gross tumour volume (GTV), PTV, organs at risk (OAR) and dose maps were present and re-named to a pre-defined convention to allow automated analysis. This was also important in facilitating comparison of dosimetry across patient databases in any future analyses [20]. Due to the size of the database, work was undertaken within the group to develop and enhance Matlab based scripts from previous published work from this group [15] to automatically process and amend the database to ensure the required uniformity. The 95 % isodose line for each patient was created and converted to a structure, V_95%_, using a Matlab script based in CERR.

### Plan quality metric

Conformity indexes are mathematical methods of quantifying the conformity of one volume with respect to another and several methods are available. A review of these methods was undertaken by Feuvret et al. [21] in which the advantages and disadvantages of a number of CIs in the context of the analysis of dose distribution are described. The Mean Distance to Conformity (MDC) index was first introduced by Jena et al. [22] to compare RT target volume delineation by different observers. For a specific volume that is being evaluated against a reference volume, the MDC represents the average distance that all outlying points in the volume must be moved in order to achieve perfect conformity with the reference volume. According to Jena, MDC provides (a) a single scoring statistic that represents the overall conformity of the two volumes being assessed, (b) additional statistics that provide information on whether the non-conformity is caused by over or under outlining and (c) a method of display that facilitates evaluation of the clinical significance of discrepancies.

Here we extend the application of MDC to dose distribution, by quantifying the conformity of the 95 % isodose line to the PTV. In our approach, the two components of the metric, OverMDC and UnderMDC, allow both ‘overdosing’ of normal tissues and ‘underdosing’ of the PTV respectively, to be objectively measured.

For the PTV and V_95%_, the MDC calculates the average distance that the furthermost outlying point of the evaluation volume (V_95%_) would have to be moved in order to conform exactly to that of the reference volume (PTV). This results in two metrics that give a measurement of the over and under coverage of the PTV by the V_95%_, known as OverMDC and UnderMDC respectively. These were calculated between the V_95%_ and PTV for each patient.

### Statistical analysis

Data analysis was undertaken in EUCLID, an outcome analysis tool for high-dimensional clinical studies based in Matlab [23], Microsoft Excel [24] and IBM’s Statistical Package for the Social Sciences (SPSS) [25].

Kaplan-Meier plots were generated to observe whether there was a threshold for the CI value in relation to survival. Outcome data for each patient, including overall survival, was collated and prepared by the WCTU. Rates of late toxicity were so low that further analysis of normal tissue effects were not pursued. Due to the adverse effect of cetuximab on survival in the SCOPE 1 trial [13], the cetuximab administration data for each patient was also acquired to allow for stratification.

Using the EUCLID package, comparisons of survival of two populations discriminated by a given variable (OverMDC and UnderMDC) was performed using a log rank test of the hypothesis that the curves describe the same survival function.

A function was used in EUCLID that allows the option to scan the range of the variable (in this case OverMDC and UnderMDC) to find the break point value that yields the lowest p-value and therefore best separates the low survival from the high survival population. This is corrected for multiple comparisons using the Bonferroni adjustment, a method of dealing with multiple testing when finding an optimal break point [26]. Cox proportional hazard, Mann-Whitney tests and stratification for cetuximab was undertaken in SPSS. Pearson tests were undertaken in Microsoft Excel.

### Effect of dose delivery method

In order to evaluate the impact of treatment delivery, we re-planned patients from 3D-conformal to Volumetric Modulated Arc Therapy (VMAT) in Oncentra OMP using a class solution, the hypothesis being that VMAT would improve the dose conformity. The class solution was developed to provide a clinically acceptable plan for the majority of patients using the VMAT technique. Any patient plans that would not meet the SCOPE 1 RT dose volume constraints following re-planning with the class solution would be adjusted manually. The dose volume constraints for the PTV and OARs were the same for every patient in the SCOPE 1 trial regardless of the dose delivery technique used (See Table 1).

**Table 1:** Dose volume constraints in SCOPE 1 trial

Region of interest/Organ at risk	Dose constraint
PTV	V95 % (47.5Gy) > 99.0 %
PTV	PTV min > 93 % (46.5Gy)
DMAX	<107 %
GTV	GTV min > 100 % (50.0Gy)
Spinal cord PRV	Cord Max <80 % (40Gy)
Combined lungs	V40 % (V20Gy) < 25 %
Heart	V80 % (V40Gy) < 30 %
Liver	V60 % (V30Gy) < 60 %
Individual kidneys	V40 % (V20Gy) < 25 %

### Multivariate analysis

Multivariate logistic regression analysis was undertaken in EUCLID using the method described by Gayou et al. [23]. The clinical variables attributable to all patients included in the analysis in addition to the cetuximab randomisation were; age, sex, tumour type (squamous cell or adenocarcinoma), tumour stage (1–4), tumour site (upper, mid, lower third), PTV volume, disease length, UnderMDC and OverMDC.

## Results

### OverMDC and UnderMDC and overall survival

**Fig. 2:**
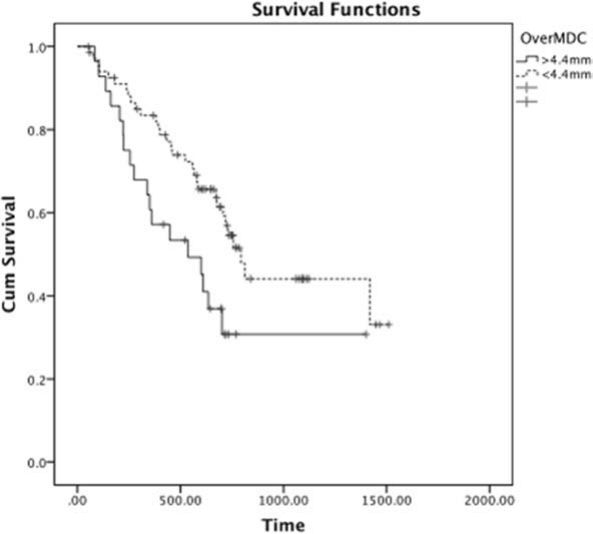
Kaplan-Meier plot of OverMDC and overall survival

For the 97 patient plans in this study, the median OverMDC was 3.7 mm (Range: 1.2–7.0 mm). The break point occurs at an OverMDC value of 4.4 mm with *p* = 0.02 (logrank) and results in a 28 above/69 below split in the database (Fig. 2).

The Cox Proportional Hazard ratio was calculated in SPSS to be 0.50 (95 % CI: 0.28–0.90, *p* = 0.02).

Stratifying for cetuximab administration, the log rank test between the two groups gave *p* = 0.04.

Therefore within this cohort, the OverMDC value for the conformity of the 95 % isodose line and the PTV for any particular patient is a predictor for overall survival in univariate analysis, independent of cetuximab administration; a high OverMDC is associated with worse survival.

### UnderMDC

**Fig. 3:**
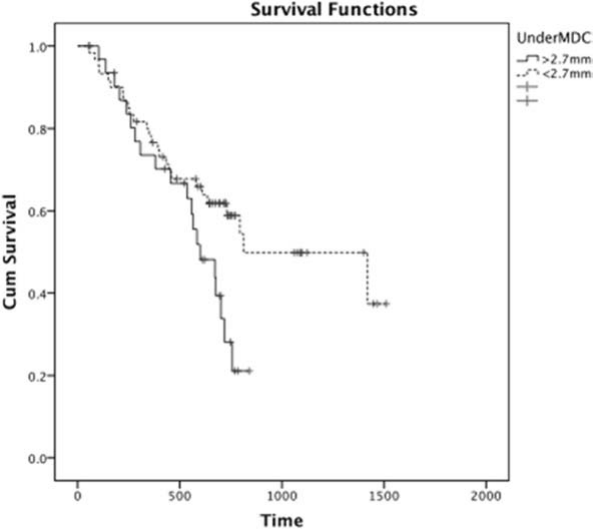
Kaplan-Meier plot of UnderMDC and overall survival in SPSS

The median UnderMDC was 2.5 mm (Range: 0–5.7 mm). The break point occurs at an UnderMDC value of 2.7 mm with a *p* = 0.05 (logrank) and results in a 32 above/62 below split in the database (Fig. 3).

The Cox Proportional Hazard ratio was calculated in SPSS to be 0.53 (95 % CI: 0.30–0.97, *p* = 0.04).

Stratifying for cetuximab administration, the log rank test between the two groups gave *p* = 0.14. Therefore UnderMDC cannot be considered a statistically significant predictor for overall survival when stratifying for cetuximab.

### Analysis of OverMDC values

As only OverMDC remained clinically and statistically significant following stratification for cetuximab, further analysis was limited to this metric. The aim was to identify factors that may influence the OverMDC calculated for each patient.

### PTV volume and OverMDC

**Fig. 4:**
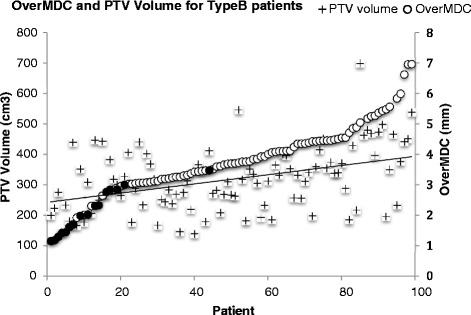
OverMDC and PTV volume values for each patient

Figure 4 shows the PTV volume for each patient plotted with OverMDC values, ranked by increasing OverMDC value, with the line of best fit for the PTV volumes. There was a significant difference in PTV volume either side of the OverMDC breakpoint of 4.4 mm (Mann-Whitney *p* < 0.001). The Pearson coefficient between the OverMDC metric and PTV volume was calculated to be 0.47.

### IMRT/VMAT dose delivery

Patients were classified according to whether the treatment dose in the SCOPE 1 trial was delivered via 3D conformal (81 patients) or IMRT/VMAT treatment (16 patients).

Figure 3 therefore also illustrates a statistically significantly lower OverMDC value if the patient was treated using IMRT/VMAT (Filled markers) compared to 3D-CRT (mean IMRT = 2.1 mm, 3D-CRT = 4.1 mm; Mann Whitney *p* < 0.001). There was no significant difference in PTV volume according to the treatment delivery method (Mann Whitney *p* = 0.455).

### Re-planning of 3D-CRT plans

The result in the previous section show the OverMDC values for 97 patients planned and treated with different treatment modalities. In order to confirm that only the treatment modality was influencing the OverMDC value, the 15 worst performing patients according to OverMDC (plotted with square symbols in Fig. 4) were re-planned from the 3D-CRT to VMAT in OMP. Ten patients were successfully re-planned using the class solution implemented in our centre. The five remaining patient plans did not meet the SCOPE 1 protocol dose volumes constraints initially, but in all cases acceptable plans were achieved after manual adjustment.

**Fig. 5:**
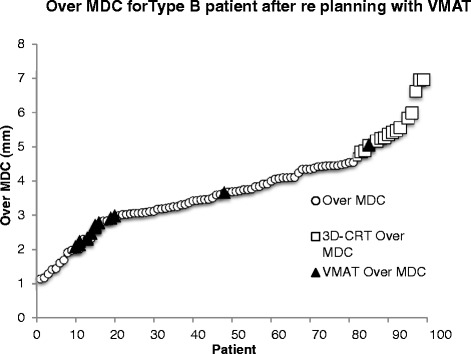
Effect of re-planning from 3D-CRT to VMAT on OverMDC values

The OverMDC was reduced for all 15 patients after replanning with VMAT (Fig. 5), with a mean reduction of 2.8 mm (Range: 1.6–4.0 mm). This confirms that the treatment modality has a large influence on OverMDC value. However there are also two outliers where the OverMDC was not reduced to the same extent. On further review it was found that these two patients had an above average PTV volume (441 cm^3^ and 498 cm^3^) when compared to the SCOPE 1 database mean PTV volume (334 cm^3^). The average PTV volume for the re-planned patients was 393 cm^3^.

### Multivariate analysis

OverMDC and UnderMDC were included in a multivariate logistic regression analysis along with other clinical factors. They were, age, sex, tumour type, tumour stage, tumour site, cetuximab administration, PTV volume and disease length. It was found that none of these factors were statistically significant. The factors with the lowest p-values were found to be age and tumour staging (*p* = 0.06 and *p* = 0.08 respectively).

## Discussion

The aim of this study was firstly to quantify plan quality using a CI and its effect on patient outcome. Secondly, to identify whether clinical and technological factors including PTV volume and treatment delivery method could be related to the CI value.

We found that OverMDC has a statistically significant relationship with overall survival in univariate analysis independent of cetuximab administration, the latter having been shown to adversely effect survival in the SCOPE 1 trial. We have also shown that PTV volume was weakly correlated with the OverMDC value of each patient (Pearson’s correlation – 0.47), but the treatment delivery method had a more significant impact with the mean IMRT OverMDC being 51 % of the mean 3D-CRT OverMDC value. When OverMDC and UnderMDC were included with other clinical variables in a multivariate logistic regression analysis neither remained significant.

The volume of the PTV may have an influence on dose coverage. Meeting constraints for larger PTVs is more difficult due to the likely increased overlap with organs at risk (OAR). Specifically in the case of oesophageal cancer, OARs such as the heart, lungs and spinal cord are in close proximity to the oesophagus and may limit the ability to optimise the dose distribution. In this study we hypothesized that a larger PTV volume would be associated with an increase in OverMDC and UnderMDC values due to the increased complexity of the resulting RT plan and ability to conform the dose to the PTV due to the need to spare adjacent OARs. Statistical tests showed that there was indeed a significant difference in the PTV volumes of patients either side of the OverMDC break point. No significant difference was found in the case of the UnderMDC metric.

This study also confirmed that IMRT and VMAT increase dose conformity when compared to 3D-conformal therapy, as shown elsewhere in the literature [27]. This is demonstrated by the significantly smaller OverMDC values between the V_95%_ and PTV volumes in the IMRT/VMAT patients of the SCOPE 1 trial and furthermore by the re-planning of the worst performing patients by OverMDC value from 3D-conformal to VMAT. A study in gastric cancers found similar results when comparing 3D-conformal radiotherapy to IMRT [28], concluding that a better target coverage and therefore significant dose reduction to OARs could be achieved in IMRT plans. It is clear therefore that the more conformal dose delivery techniques should be used to administer RT wherever possible.

The explanation for the improved overall survival in patients treated with a lower OverMDC value more conformal treatment is not clear. The association with IMRT/VMAT treatment is interesting as only 3 centres treated the 16 patients with IMRT/VMAT in the original trial. In addition Fig. 5 clearly shows two cases where re-planning with VMAT/IMRT did not reduce the OverMDC value to the same extent, but their PTV volume was higher than the average for the patients included in this study. It is possible that low OverMDC and/or access to IMRT/VMAT reflect other aspects of a high quality RT process that require further investigation in a future study. It is also fully acknowledged that when the OverMDC metric is included in a multivariate analysis that includes other common factors in cancer treatment, it does not remain statistically significant. Furthermore, there was no recorded grade 3 or 4 toxicity for pneumonitis or of the heart most commonly associated with radiotherapy of the oesophageous in the SCOPE 1 trial. As a result, no meaningful correlation between dose distribution and radiotherapy induced toxicity can be made. It may be therefore that the consequence of a plan’s OverMDC value on overall survival is a combination of a number of factors that require further investigation outside the scope of this particular study.

Nevertheless, this study suggests that IMRT/VMAT offers a safe tool for dose escalation, as the MDC analysis has shown that unnecessary irradiation of normal tissue can be significantly reduced without affecting PTV coverage. This is consistent with the findings of Freilich and Chun et al. [29, 30] who concluded that although the use of IMRT did not directly impact on survival, it was associated with significantly less toxicity. Warren et al. also showed that IMRT allows dose escalation to 60Gy with the same level of normal tissue irradiation as 3D-CRT to 50Gy [8]. This is being taken forward in the recently funded SCOPE 2 trial. Unfortunately there were an insufficient number of patients treated with IMRT/VMAT in this study to detect any impact on survival. In addition, patients treated with IMRT/VMAT may have been expected to have a lower rate of toxicity, however the rates were so low within the SCOPE 1 trial that this could not be studied.

In a clinical setting, the results of this study suggest that careful attention to the quality of RT planning, expressed in terms of conformity of the dose distribution to the target volume, may impact on overall survival. IMRT/VMAT should be considered for all patients when conforming the 95 % isodose to the PTV is difficult.

## Conclusion

We have shown using a CI that in univariate analysis the quality of a plan with respect to PTV coverage has a significant correlation with overall survival. Plan quality is strongly related to the use of advanced RT delivery techniques and to a lesser extent with PTV volume. A CI may therefore be useful in assessing plan quality and we recommend careful attention to all aspects of PTV coverage.

## Abbreviations

dCRT: Definitive chemoradiotherapy
RT: Radiation therapy
PTV: Planning treatment volume
RTTQA: Radiotherapy trials quality assurance
CI: Conformity index
MDC: Mean distance to conformity
WCTU: Wales Cancer Trials Unit
CERR: Computational Environment for Radiotherapy Research
GTV: Gross tumour volume
OAR: Organ at risk
VMAT: Volumetric Modulated Arc Therapy
SPSS: Statistical Package for the Social Sciences
IMRT: Intensity modulated radiation therapy
